# Impact of corticosteroid administration on the response of exposed dental pulp to capping with bioactive cements-experimental study on mongrel dogs

**DOI:** 10.1186/s12903-023-03119-3

**Published:** 2023-06-26

**Authors:** Hanan A. Soliman, Radwa Ibrahim EL-Toukhy, Mona Mohsen Abdo Ibrahim, Mohammed E. Grawish, Mohamed Abdel kader Sobh, Salah Hasab Mahmoud

**Affiliations:** 1 Conservative Dentistry Department, Faculty of Dentistry, Kafrelsheikh University, Kafrelsheikh, Egypt; 2 Conservative Dentistry Department, Faculty of Dentistry, Mansoura University, Mansoura, Egypt; 3 Oral Pathology Department, Faculty of Dentistry, Mansoura University, Dakahlia Governorate, Egypt; 4 Oral Biology Department, Faculty of Dentistry, Mansoura University, Dakahlia Governorate, Egypt; 5 Oral Biology Department, Faculty of Oral and Dental Medicine, Delta University for Science and Technology, Dakahlia Governorate, Egypt; 6 Nephrology Department, Urology and Nephrology Center, Faculty of Medicine, Mansoura, Egypt; 7 Conservative Dentistry Department, Faculty of Dentistry, Horus University, New Damietta City, Egypt; 8 Conservative Dentistry Department, Faculty of Dentistry, Mansoura University, Mansoura, Egypt; 9 Oral Pathology Department, Faculty of Dentistry, Mansoura University, Mansoura, Egypt

**Keywords:** Dogs’ teeth, Corticosteroid, Pulp capping material, Direct pulp capping technique

## Abstract

**Background:**

Corticosteroids are commonly used as a treatment for a variety of pathological conditions, however, systemic corticosteroid administration has adverse effects including impaired immune response and wound healing. Such complications may affect pulp healing after direct pulp capping. The current study evaluated the influence of corticosteroids on the healing ability of exposed dogs’ dental pulps after direct pulp capping (DPC) with bioactive materials.

**Methods:**

Ten healthy male dogs were assigned randomly into two groups, 5 dogs each: group I represent the control group which did not receive any medication, and group II was given corticosteroid for 45 days before DPC and till the dogs were euthanized (n = 75 teeth for each group). Following mechanical exposure, the pulps were randomly capped with either Ca(OH)_2,_ MTA, or Biodentine. The pulpal tissues’ reaction to the capping materials was evaluated 65 days postoperatively according to the following parameters: calcific bridge formation, pulpal inflammation, pulp necrosis, and bacterial infiltration.

**Results:**

The corticosteroid-treated group revealed no significant difference compared to the control group concerning the pulp healing response (*P* > 0.05). Both Biodentine and MTA-treated specimens revealed significant differences with Ca(OH)_2_-treated specimens (*P* < 0.05) which displayed a superior positive effect of both MTA and Biodentine to Ca(OH)_2_ regarding all the parameters.

**Conclusions:**

Direct pulp capping technique whenever indicated in subjects treated with corticosteroid immunosuppressive drugs like prednisone performed well in aseptic conditions especially when capped with bioactive materials.

## Background

It is crucial to preserve the vitality of dental pulp that is exposed by iatrogenic causes, via instrumentation or dental trauma. Direct pulp capping (DPC) is practiced as a conservative therapy of traumatic pulp exposure by applying a protective material to salvage vital pulp. Regarding the scientific overview of DPC, effective pulp capping material should have a good sealing ability, bioinductivity, biocompatibility, and should be insoluble and have antibacterial effects [1].

Calcium hydroxide-based cement [Ca(OH)_2_] is one of the most common agents broadly applied in DPC and it had been considered as the gold standard up till 1999. These types of cement were considered to have drawbacks such as improper dentin adhesion, dissolution in tissue fluids, porous organization, and inability to withstand stresses under tooth flexure [2]. Nowadays, bioactive materials such as calcium silicate-based materials (CSCs), including mineral trioxide aggregate (MTA) and calcium silicate-based cement (Biodentine) have attracted more attention as efficient alternative materials for Ca(OH)_2_ due to their higher clinical success [3].

Biomaterials are not the only factors that can influence the prognosis of DPC. Factors such as age, systemic health, systemic drugs, and clinical signs including type, size, and extent of the pulp exposure, pulpal hemorrhage, and proper restoration also interfere with the success of DPC. Regarding the systemic condition and the use of drugs, inflammatory autoimmune diseases and severe infections are treated with the systemic use of corticosteroids [4].

Nevertheless, the healing capacity of the exposed dental pulp in patients receiving corticosteroids is still a debatable issue. Corticosteroid agents may suppress local pulp repair as well as inflammation [5]. This resulted from inhibition of matrix metalloproteinases (MMPs) [6] which are produced during pulp tissue inflammation to stimulate fibroblast wound healing, angiogenesis and reparative dentin formation [7]. Studies reported that corticosteroids impact wound healing, [8] whereas others reported a successful tissue healing following systemic corticosteroid treatments [9, 10]. Animal studies are recommended to assess the efficacy of pulp-capping materials regarding to ISO 7405:2008 for dentistry [11]. This study aimed to investigate the pulpal tissue response after DPC with Ca(OH)_2_, MTA or Biodentine of mechanically exposed teeth in corticosteroid-treated dogs. The research null hypothesis was that there was no difference in pulp healing response of mechanically exposed dental pulp capped with different capping materials regarding calcific bridge formation, pulpal inflammation and pulpal necrosis in control and corticosteroid-treated groups.

## Methods

### Sample size calculation

The sample size of this animal study was statistically determined using G* Power 3.1.9.2 software. The statistical model selected was X [2] and the statistical test was goodness-of fit tests: Contingency tables (Chi-squared test). A priori: compute required sample size was used as the type of power analysis given effect size, power, and α. The input parameters were medium effect size (0.3), an error probability (α) of 0.05, a power of 0.95 and number of groups was 2 [12]. The estimated sample size was 145, thus the total number was 150 teeth for adequate division of this number by 6 without reminders (25 teeth/ each material/ 75 teeth/ group).

### Materials

The materials tested in the current study, their composition, manufacture and the lot number are presented in Table 1.

**Table 1:** Materials used in the current study

Materials	Composition (by wt)	Manufacture	Lot no.
**Calcium Hydroxide based cement (DYCAL)**	**Base paste**: Disalicylate ester of 1,3, butylene glycol <50% Calcium tungstate 20% Zinc oxide <15% **Catalyst paste**: Ca(OH)_2_ <55% Titanium dioxide (radiopacifiers) 10% Zinc oxide <15	DYCAL ,DENTSPLY caulk, Milford,DE, USA	160,801
**Mineral trioxide aggregate** **(MTA)**	**Powder**: Calcium Carbonate 60–80% Silicon dioxide 5–15% Aluminum oxide (radiopacifiers) 5–10% Calcium zirconia complex 20–30% **Liquid**: distilled water	RetroMTA, BioMTA, Seoul, Korea	1509D04
**Tricalcium silicate-based cement capsule** **(Biodentine )**	**Powder**: Tricalcium silicate (main core material) 80,1% Dicalcium silicate (second core material) Calcium carbonate and oxide (fillers) 15% Iron oxide shade( coloring agent) Zirconium oxide (radiopacifiers) 5% **liquid**: Calcium Chloride (accelerator) Hydrosoluble Polymer: modified poly carboxylate	Septodont, St, Maur-des-Fosses, France	B25134
**Resin modified Glass Ionomer Cement capsule** **(RGIC)** **(Shade A3)**	**Powder**: Alumino-Fluoro-silicate glass **Liquid**: 2-hydroxyethyl methacrylate (HEMA) 25–50% polybasic carboxylic acid** 5–10% dimethacrylate (UDMA) 1–5% dimethacrylate** 1–5%	Fuji II LC; GC Corporation, Tokyo, Japan	1,904,191

### Animal selection and study design

The Medical Research Ethics Committee at Delta University approved the current study based on a protocol no. (FODMRC-2022-00105). The manuscript of this animal study has been written following the items listed in the ARRIVE guidelines 2.0 [13]. Ten healthy male mongrel dogs aged 2–3 years, weighing 15-20 kg were randomly selected from the animal house using a random number generator [13]. The dogs were free of any systemic diseases and kept under observation for 2 weeks of quarantine by an expert veterinarian before starting the operative procedures.

The dogs were classified in two groups; group I represent control group which did not receive any drugs, but those of group II were given corticosteroid (Solupred, Prednisolone, Sanofi-Aventis, France) in a dose of 1 mg/ kg/ day [14, 15]. The orally administered drugs were preoperatively taken for 45 days and till the dogs were euthanized. For each dog, class V cavities were prepared on the labial surfaces of 15 anterior teeth. The pulp was mechanically exposed and capped with either Ca(OH)_2_ (DYCAL ,DENTSPLY caulk, Milford,DE, USA), MTA (RetroMTA, BioMTA, Seoul, Korea) or Biodentine (Septodont, St, Maur-des-Fosses, France) randomly using ‘Research Randomizer‘ (www.randomizer.org) to each set of teeth, then sealed by RGIC (Fuji II LC; GC Corporation, Tokyo, Japan) which can be applied over freshly mixed MTA with minimal effects on the MTA [16]. The evaluation of the pulp capping effect on pulp healing was carried out 65 days postoperatively.

### Experimental procedures

Each dog was intravenously injected with 6 mg/ kg thiopental sodium (EIPICO, 10th of Ramadan, Egypt) on the day of the operation. Accordingly, general anaesthesia was controlled using a concentration of 1.5–2.5 halothane (ACDIMA, 6th October, Egypt) in oxygen, delivered through a semi closed breathing circuit [17].

When polishing and isolation of teeth finished, standard class V cavities were prepared on the labial surfaces of each tooth with dimensions of 2.5, 3, and 1.5-2 mm for width, length, and depth, respectively using a tungsten carbide pear-shaped bur #500 314 139,008 009 (Komet, Lemgo, Germany) at high speed with oil free air/ water cooling. For every four cavities, a new bur was used and cavities were cut 0.5-1 mm above the free gingiva. The dental pulp was exposed with a sterile sharp endodontic explorer (DG16, Dental USA Inc., Mc Henry, IL, USA).

Before application of pulp capping materials, pulpal hemorrhage was controlled by pressing with a sterile saline-soaked cotton pellet [18]. Consequently, RGIC, a sealing material, was used to fill each cavity [19]. All capping and sealing materials were prepared and applied according to manufacturer instructions.

To minimize the pain and discomfort, dogs were given soft food and analgesics. They were kept in good, ventilated cages and were continuously observed by an expert veterinarian for 65 days. Then, the dogs were sacrificed using overdose injection of pentobarbital sodium at the end of the experimental period [20]. A hand saw was used to separate each anterior tooth of the upper and lower jaws into individual blocks. Thereafter, removal of the apical portion of the roots before teeth immersion in 10% neutral buffered formalin solution was performed for 7 days. Later, teeth were decalcified using a changed daily 10% EDTA solution for 6 month [21].

After embedding in a paraffin wax, teeth were sectioned at an average thickness of 5 μm in a labio-lingual direction. Finally, for histo-microbiological analyses, the slides were stained with hematoxylin-eosin (H&E) and modified Brown-Brenn stains [22]. To avoid any possible bias, two blinded expert examiners investigated all the specimens Inter- and intra-examiner reliability was assessed using Kappa statistics. (Cohen kappa ≥ 0.8). For each serial sections, 5 measurements were taken and classified depending on the following parameters: calcific bridge formation, pulpal inflammation, pulp necrosis, and bacterial infiltration [14, 23].

Calcific bridge formation: It was classified as none: no hard tissue formation; initial: limited hard tissue deposition below or around the exposure, extending to no more than half of the exposure site; partial: hard tissue deposition below or around the exposure, extending to more than half but not completely closing the exposure site; and complete: hard tissue formed across the exposure site completely.

Pulpal inflammation was classified as none: absence of inflammatory cells; slight, a few scattered inflammatory cells; moderate: moderate inflammatory cell infiltration around the exposure site; and severe: heavy inflammatory cell infiltration of the coronal pulp.

Tissue necrosis: It was classified as none: presence of complete tissue organization in the entire pulp; mild: necrosis was close to the pulp exposure site with normal central pulp; moderate: more widespread disorganization of the pulp tissue morphology; and severe: complete tissue necrosis of the pulp. Bacterial infiltration: positive bacterial infiltration was characterized by the presence of bacteria in the pulp space, the axial floor, or along the cavity walls and within the cut dentinal tubules.

### Statistical analysis

SPSS program, version 26.0, was used to organize, code, and analyse the data. The Kruskal Wallis statistical test was utilized for total comparison. Then, Mann-Whitney U test was used for pairwise comparison. Statistically significant differences were regarded at *P* < 0.05.

## Results

**Fig. 1:**
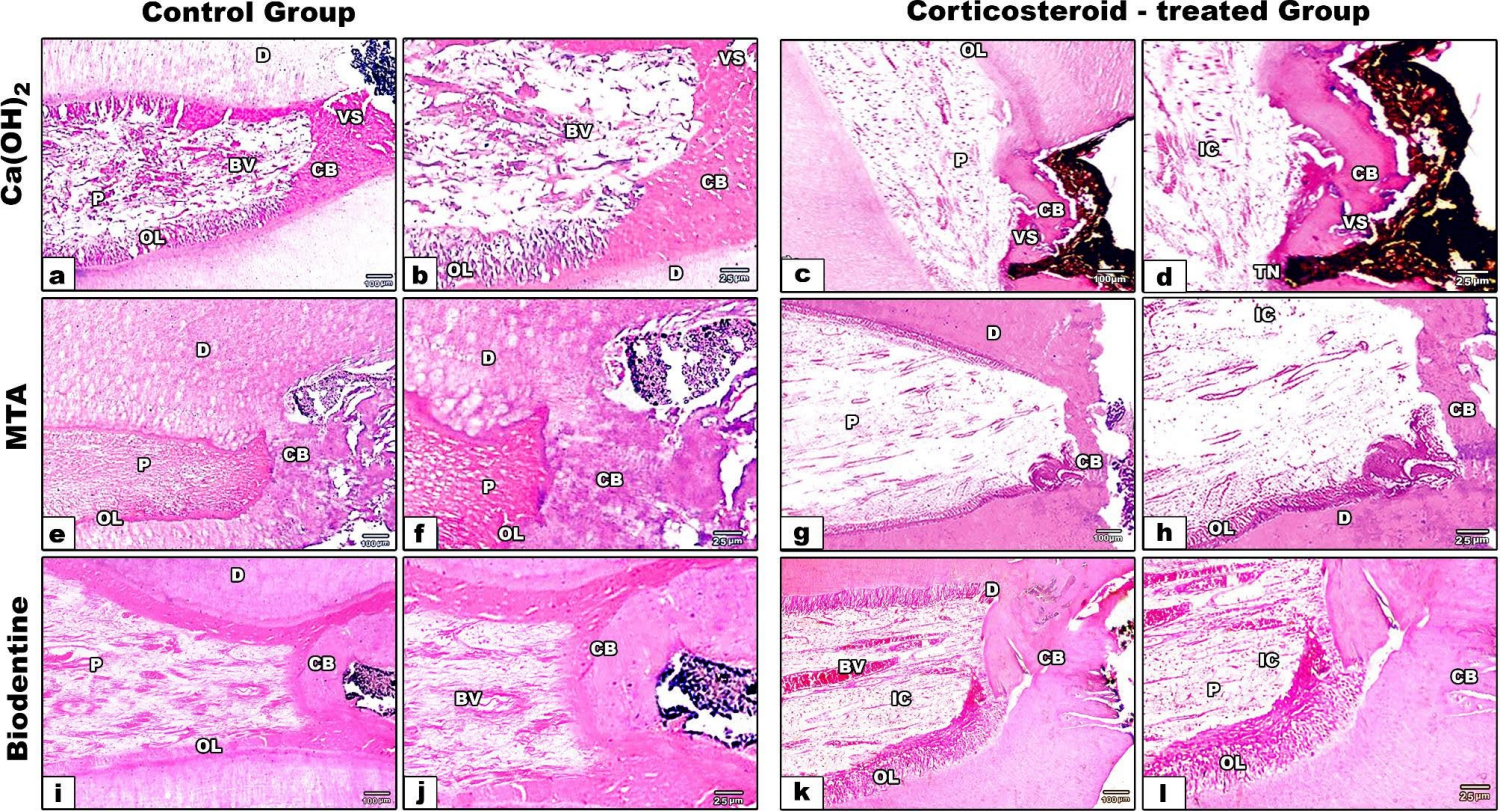
Photomicrographs of decalcified sections of the control and corticosteroid- treated groups at 65 days after pulp exposure allocated according to the materials used. **a,b,c &d**; Ca(OH)2, **e,f,g &h**; MTA and **I,j,k&l;** Biodentine. Ca(OH)2-treated specimens show reparative CB with VS and normal pulp with mild IC in the control group (**a&b**);however those in corticosteroid-treated group show partial CB with mild pulp disorganization underneath the capping material at the site of pulp exposure (**c&d**). MTA-treated specimens in both control and corticosteroid- treated groups show complete CB with normal tissue organization (**e,f,g&h**), however corticosteroid -treated group shows mild IC (**g&h**). Biodentine-treated specimens show thick CB and normal pulp organization with mild IC in the control group (**i&j**) and moderate IC in corticosteroid-treated specimens (**k&l**) CB, calcific bridge; VS, vacuole-like structure; IC, inflammatory cell infiltrates; D: dentin; OL: odontoblast cell layer; P: pulp; BV: blood vessels; Arrow heads show exposure sites; (H&E, a,c,e, g,i &k; 100x,/b,d, f, h, j& l 400x)

**Fig. 2:**
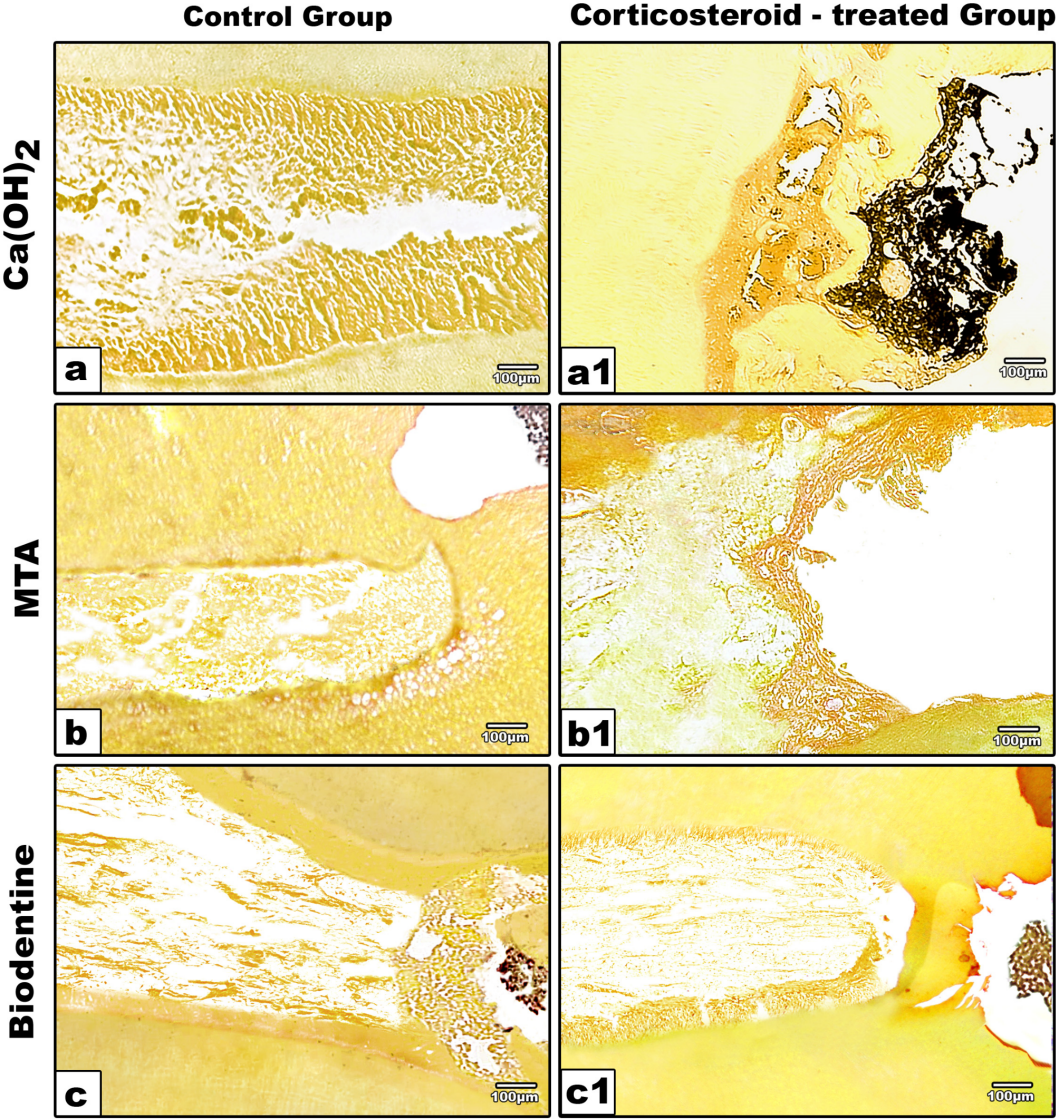
Photomicrograph of decalcified section of the control and corticosteroid- treated groups at 65 days after pulp exposure stained with Brown-Brenn allocated according to the materials used. **a,a1**; Ca(OH)2, **b,b1**; MTA and **c,c1** Biodentine. No positive bacterial infiltration shown in control group specimens(a,b,c) and in corticosteroid-treated group (a1,b1,c1)(Brown-Brenn,100x).

Histologic evaluation of teeth after the stipulated time of 65 days showed that the pulp tissues were well tolerated for MTA and Biodentine in most specimens of control and corticosteroid-treated groups. Ca(OH)_2_-treated specimens revealed partial to complete calcific bridge formation with minimal to moderate pulpal inflammation, in addition to none/ moderate pulp disorganization in control group (Fig. 1.a & b). While Ca(OH)_2_-treated specimens of corticosteroid-treated group showed incomplete bridge formation with minor defects of cell inclusions in addition to no to moderate pulpal necrosis and minimal to moderate pulpal inflammation (Fig. 1. c & d). The presence of pulpal inflammation in Ca(OH)_2_-treated specimens was represented by the presence of obvious dilated blood vessels and diffuse inflammatory cell infiltrates. Ca(OH)_2_-treated specimens exhibited a lower incidence of calcific bridge formation than other materials with increased formation of vacuole like structure in control (Fig. 1. a & b) and corticosteroid-treated (Fig. 1.c & d) groups. Ca(OH)_2_ particles were embedded in the newly formed calcific bridge (Fig. 1.c & d). MTA and Biodentine-treated specimens show partial to complete calcific bridge formation and no to slight pulpal inflammation with normal pulp organization in both control (Fig. 1.e, f, i, j), and corticosteroid-treated groups (Fig. 1.g, h, k & l). No bacterial infiltration was recognized in all the specimens of both control (Fig. 2. a,b,c) and corticosteroid-treated groups stained with modified Brown – Brenn stain (Fig. 2. a1,b1,c1). Both Biodentine and MTA -treated specimens revealed significant differences with Ca(OH)_2_-treated specimens, although no significant differences were found between Biodentine and MTA-treated specimens (Tables 2 and 3). Regarding all variables, results demonstrated no statistically significant difference between corticosteroid-treated and control groups (U test, *P* > 0.05) (Table 4).

**Table 2:** Score percentages of calcific bridge formation, pulpal inflammation and tissue necrosis in the control group after DPC with Ca(OH)2 based cement, MTA and Biodentine and their statistical analyses

Parameter	Pulp capping materials					
	Scores		Ca(OH)_2_ based cement	MTA	Biodentine	P†
**Calcific bridge formation**	Percentage	None	0.0%	0.0%	0.0%	< 0.05*
		Initial	37.5%	0.0%	0.0%	
		Partial	50.0%	25.0%	12.5%	
		Complete	12.5%	75.0%	87.5%	
	Median (range)		3.0 (2.0–4.0)	4.0 (3.0–4.0)	4.0 (3.0–4.0)	
	P‡			P1 = 0.007*	P1 = 0.004* P2 = 0.84	
**Pulpal inflammation**	Percentage	Minimal	25.0%	87.5%	87.5%	< 0.05*
		Mild	50.0%	12.5%	12.5%	
		Moderate	25.0%	0.0%	0.0%	
		Severe	0.0%	0.0%	0.0%	
	Median (range)		2.0 (1.0–3.0)	1.0 (1.0–2.0)	1.0 (1.0–2.0)	
	P‡			P1 = 0.04*	P1 = 0.04* P2 = 1.00	
**Tissue necrosis**	Percentage	None	37.5%	100.0%	100.0%	< 0.05*
		Mild	37.5%	0.0%	0.0%	
		Moderate	25.0%	0.0%	0.0%	
		Severe	0.0%	0.0%	0.0%	
	Median (range)		2.0 (1.0–3.0)	1.0 (1.0–1.0)	1.0 (1.0–1.0)	
	P‡			P1 = 0.04*	P1 = 0.04* P2 = 1.00	

^†^Kruskal Wallis, ^‡^Mann-Whitney U, P1: significance Vs Ca(OH)_2_ based cement subgroup and P2: significance Vs MTA subgroup *Significant at ≤ 0.05

**Table 3:** Score percentages of calcific bridge formation, pulpal inflammation and tissue necrosis in the corticosteroid -treated group after DPC with Dycal, MTA and Biodentine and their statistical analyses

Parameter		Pulp capping materials							
		Scores				Ca(OH)_2_ based cement	MTA	Biodentine	*P* ^†^
**Calcific bridge formation**	Percentage		None			12.5%	0.0%	0.0%	< 0.001*
			Initial			50.0%	0.0%	0.0%	
			Partial			37.5%	50.0%	37.5%	
			Complete			0.0%	50.0%	62.5%	
	Median (range)				2.0 (1.0–3.0)		3.5(3.0–4.0)	4.00(3.0–4.0)	
	*P* ^‡^						*P*1 = 0.002*	*P*1 = 0.001* *P*2 = 0.77	
**Pulpal inflammation**	Percentage		Minimal	25.0%			75.0%	87.5%	< 0.05*
			Mild	50.0%			25.0%	12.5%	
			Moderate	25.0%			0.0%	0.0%	
			Severe	0.0%			0.0%	0.0%	
	Median (range)			2.0 (1.0–3.0)			1.0 (1.0–2.0)	1.0(1.0–2.0)	
	*P* ^‡^						*P*1 *=* 0.04*	*P*1 = 0.015* *P*2 = 0.7	
**Tissue necrosis**	Percentage		None	25.0%			87.5%	87.5%	< 0.05*
			Mild	62.5%			12.5%	12.5%	
			Moderate	12.5%			0.0%	0.0%	
			Severe	0.0%			0.0%	0.0%	
	Median (range)			2.0 (1.0–3.0)			1.0(1.0–2.0)	1.0(1.0–2.0)	
	*P* ^‡^						*P*1 = 0.02*	*P*1 = 0.02* *P*2 = 1.00	

^†^Kruskal Wallis, ^‡^Mann-Whitney U, P1: significance Vs Ca(OH)_2_ based cement subgroup and P2: significance Vs MTA subgroup *Significant at ≤ 0.05

**Table 4:** Results of statistical analysis between different pulp capping materials (Ca(OH)2 based cement, MTA and Biodentine) regarding calcific bridge formation, pulpal inflammation and tissue necrosis in control and corticosteroid-treated groups

Material	Parameter	Scores	Control Group	Corticosteroid - treated Group	*P* ^*†*^
**Ca(OH)**_**2**_ **based cement**	Calcific bridge formation	Median (Range)	3.0 (2.0–4.0)	2.0 (1.0–3.0)	< 0.05^*^
		*P* ^‡^	0.23		
	Pulpal inflammation	Median (Range)	2.0 (1.0–3.0)	2.0 (1.0–3.0)	
		*P* ^‡^	1.00		
	Tissue necrosis	Median (Range)	2.0 (1.0–3.0)	2.0 (1.0–3.0)	
		*P* ^‡^	0.98		
**MTA**	Calcific bridge formation	Median (Range)	4.0 (3.0–4.0)	3.5 (3.0–4.0)	< 0.05^*^
		*P* ^‡^	0.49		
	Pulpal inflammation	Median (Range)	1.0 (1.0–2.0)	1.0 (1.0–2.0)	
		*P* ^‡^	0.7		
	Tissue necrosis	Median (Range)	1.0 (1.0–1.0)	1.0 (1.0–2.0)	
		*P* ^‡^	0.69		
**Biodentine**	Calcific bridge formation	Median (Range)	4.0 (3.0–4.0)	4.00 (3.0–4.0)	< 0.05^*^
		*P* ^‡^	0.44		
	Pulpal inflammation	Median (Range)	1.0 (1.0–2.0)	1.0 (1.0–2.0)	
		*P* ^‡^	1.00		
	Tissue necrosis	Median (Range)	1.0 (1.0–1.0)	1.0 (1.0–2.0)	
		*P* ^‡^	0.7		

^†Kruskal Wallis, ‡Mann−Whitney U, *Significant at ≤ 0.05^

## Discussion

Whenever trauma or iatrogenic damage results in pulp exposure, capping of the exposed healthy dental pulp is targeted to preserve the pulp vitality. The successful DPC therapy is not only restricted to clinical signs and symptoms [24] including pulpal hemorrhage, that is a clinical indicator of pulpal inflammation, [18] but also bioactivity of pulp capping materials and the capability of final restorative materials to provide coronal seal .

In addition, the role of general health should be taken into consideration to achieve a successful DPC technique [25]. Corticosteroids are primary stress hormones that adjust a variety of physiologic processes and are critical for life [4]. Synthetic corticosteroids are one of the most commonly prescribed drugs in the world and represent one of the most effective clinical treatments for a range of inflammatory and autoimmune diseases, such as asthma, septic shock rheumatoid arthritis, allergy, multiple sclerosis, severe coronavirus disease 2019 [4, 26]. Information about the effect of the corticosteroids on the pulpal healing response is controversial. Therefore, this animal model was performed to assess the effects of corticosteroids on pulp healing in patients who received long-term corticosteroids and sought dental treatment.

The rationale behind using dogs as an animal model was due to their similar mechanism of reparative dentinogenesis to that of human being, but with a rapid rate [27]. Besides, the dogs have adequate pulp size for the histopathological evaluation and provide a good number of teeth allowing the comparison of the pulpal response toward CSCs versus Ca(OH)_2_ as a control material in the same dog. The major objective with the use of restorative materials is to provide tight coronal seal to avoid microbial contamination therefore, RMGIC has good sealing properties, and thus it was used in this present study to inhibit microleakage leading to bacterial infection that reduces pulp healing [19].

The true “gold standard” of pulp status evaluation is the histological assessment. Most of the studies that included histological assessments were of quite a short duration [28]. In the current study, the dogs were euthanized after 65 days to produce a sufficient time for bridge formation at the cavity floor [29].

According to the histopathological analysis of the current study, positive formation of dentin bridges in corticosteroid-treated groups was observed. The formed calcific bridges were thinner and less homogenous with minor defects of cell inclusions than those of control group which might be related to the dose and duration of the administrated drug. This finding came in contrast to many researchers who reported an adverse impact of corticosteroids upon pulp healing and reparative dentin formation [5–8]..Meanwhile, many previous animal studies complied with the current finding and supported the role of systemic corticosteroids in active formation of calcific bridges and reparative dentinogenesis [9, 10, 15].Corticosteroids are the most effective anti-inflammatory therapy that reduce the production of chemicals that cause inflammation. Alliot-Licht et al. stated that corticosteroids intensely stimulated alkaline phosphatase activity and induced the expression of the transcript encoding the major odontoblastic marker, thus these medications induce mineralization [9].This mechanism could explain the positive pulpal tissue response of corticosteroid -treated group in this animal study. Nevertheless, the results indicated no statistically significant difference between control group and corticosteroid treated group in relation to calcific bridge formation, pulpal inflammation, and pulp necrosis. This statistical result might vary with different sample sizes. Additional parameter concerning the calcific bridge thickness would be considered in future studies.

In the present study, all tested materials represent a biocompatible behaviour to that productive cells could attach and produce fibrous connective tissue for further mineralization and calcified bridge formation. However, there was an adverse result clearly observed in Ca(OH)_2_-treated specimens compared with MTA and Biodentine (CSCs) in relation to calcific bridge formation, pulpal inflammation, and pulp necrosis, in both the control and corticosteroid-treated groups. This result may be related to the stronger immunoreactivity of Dycal in comparison to CSCs [2].Additionally, most Ca(OH)_2_-treated specimens in both groups revealed the formation of tunnel defects within the calcific dentin bridges this might be due to the absence of adhesion between Ca(OH)_2_ and dentin. Thus, the particles might migrate into the pulp tissue forming tunnels through dentinal bridges which act as pathways for microleakage. As well as Ca(OH)_2_ dissolve over time [30].The results of the present study were similar to those reported in previous studies which stated that hydraulic calcium silicate-based cements as MTA and Biodentine, showed lower toxicity and better biocompatibility and bioactivity than a non-silicate-based cement (i.e. Dycal) [31].

This study displayed that both MTA and Biodentine were superior to Ca(OH)_2_ regarding all the parameters. Such result can be explicated by their bio-inductivity as these materials stimulate an early reparative dentin formation [32].MTA and Biodentine release Ca(OH)_2_ as a by-product, but unlike pure Ca(OH)_2_, that dissolves over time. Thus, they are likely able to seal the exposed pulpal tissue [33].Moreover, they promote secretion of pro‑inflammatory cytokines which affects the odonto/osteogenic differentiation of stem cells [34].

Conversely, the calcific bridge thickness was relatively increased in the Biodentine group that may be related to the different velocity of the setting process of both materials. Biodentine has a faster setting time than MTA due to adding calcium chloride and calcium carbonate. Furthermore, the adhesion of Biodentine to dentin is basically micromechanical by the formation of “mineral infiltration zone” [35].whereas, MTA bonds chemically to dentin surface by formation of hydroxyapatite at MTA-dentin interface. Thus, Biodentine has an increased strength during the early phase, increased resistance toward washout and superior sealing properties than MTA [36].

The setting reaction of CSCs, including Biodentine and MTA, results in the formation of Ca (OH)_2_ that dissociates in the presence of moisture to hydroxyl ions and calcium ions that promote material bioactivity and apatite layer formation [37]. The hydroxyl ions are responsible for the increased alkalinity and antibacterial activity. Consequently, this high alkalinity initiates an inflammatory rupture, immune cells and the pulpal stem cells endured differentiation toward an osteoblast-like phenotype for the formation of a calcific bridge [38].These findings are similar to that of the study by Kassis et *al* that investigated the response of dental pulp capped with calcium-silicate based material, calcium hydroxide and adhesive resin in rabbit teeth. This study revealed a thicker hard tissue barrier formed in exposed dental pulp treated with Biodentine than other materials [39].

The major limitation of the present study is the shorter period of 65-days. Therefore further studies with longer follow-up periods and using additional parameter concerning the calcific bridge thickness may lead to more predictable results as pulpal inflammation decreases over time, thus dentin bridge thickness increases simultaneously [40, 41].These studies are relevant for patients who received long-term corticosteroids and subjected only to small exposures created mechanically during dental treatment and without caries.

Considering the above-mentioned findings, the null hypothesis of no differences in the pulp tissue response with the use of corticosteroids in dogs’ exposed pulp capped with CSCs can be accepted. The potential clinical relevance of these results is that stringent aseptic DPC of mechanically exposed pulpal tissues using bioactive materials such as Biodentine and MTA seems to be a suitable technique for conserving pulp vitality in patients receiving corticosteroids.

## Data Availability

All data generated or analyzed during this study are included in this published article.
